# Dupuytren Disease: Prevalence, Incidence, and Lifetime Risk of Surgical Intervention. A Population-Based Cohort Analysis

**DOI:** 10.1097/PRS.0000000000009919

**Published:** 2022-11-22

**Authors:** Dieuwke C. Broekstra, Rachel Y. L. Kuo, Edward Burn, Daniel Prieto-Alhambra, Dominic Furniss

**Affiliations:** Oxford, United Kingdom; and Groningen, The Netherlands; From the 1 Nuffield Department of Orthopaedics, Rheumatology, and Musculoskeletal Sciences, University of Oxford, Botnar Research Centre; 2 Department of Plastic Surgery, University of Groningen, University Medical Center Groningen; 3 Department of Plastic and Reconstructive Surgery, Oxford University Hospitals NHS Foundation Trust, John Radcliffe Hospital.

## Abstract

Health care burden attributable to Dupuytren disease (DD) is largely unknown. The authors determined (1) the prevalence and incidence of DD, (2) the incidence of first surgical intervention, and (3) the lifetime risk of surgical intervention in the United Kingdom National Healthcare Service.

**Methods:**

In this population-based dynamic cohort analysis, data of the Clinical Practice Research Datalink was linked to Hospital Episode Statistics, to characterize the diagnosis and surgical treatment of DD. Secular trends of incidence of DD diagnosis and first surgical treatment were calculated for 2000 to 2013. A multistate Markov model was designed to estimate the lifetime risk of first surgical intervention.

**Results:**

A total of 10,553,454 subjects were included in the analyses, 5,502,879 (52%) of whom were women. Of these, 38,707 DD patients were identified. Point prevalence in 2013 was 0.67% (99% CI, 0.66 to 0.68). The incidence of DD almost doubled from 0.30 (99% CI, 0.28 to 0.33) per 1000 person-years in 2000, to 0.59 (99% CI, 0.56 to 0.62) per 1000 person-years in 2013. The incidence of first surgical intervention similarly increased from 0.29 (99% CI, 0.23 to 0.37) to 0.88 (99% CI, 0.77 to 1.00) in the same period. A man or woman newly diagnosed with DD at age 65 has a lifetime risk of surgical intervention of 23% and 13%, respectively, showing only a very subtle decrease when diagnosed later in life.

**Conclusions:**

DD is an important health condition in the older population, because prevalence and incidence rates have almost doubled in the past decade. Estimated lifetime risk of surgical treatment is relatively low, but almost twice in men compared with women.

**CLINICAL QUESTION/LEVEL OF EVIDENCE:**

Risk, III.

Dupuytren disease (DD) is the most common organ-specific fibrotic disease.^1^ Patients develop nodules in the palmar side of the hand, which can progress into cords and may lead to finger contractures. These contractures lead to problems with hand function and interfere with activities of daily living.^2–4^ The disease occurs more frequently in men.^5^ The cause is multifactorial: there is a strong familial component,^6–8^ and several nongenetic associations, including exposure to smoking, alcohol intake, and diabetes.^9^ Current treatment is focused on relieving symptoms, and finger contractures are mostly treated by surgical division or excision of the cords. Unfortunately, the self-reported functional outcome after surgical treatment is poor in 22% to 59%.^10^ The mean annual costs of surgical treatment for DD in the United Kingdom (UK) are £3100 per patient, with higher costs for revision surgery.^11^ Because the disease tends to recur after treatment and the number of patients needing revision surgery is increasing,^11,12^ it is also expected that the costs of management of DD will increase.

Reported prevalence rates of DD vary widely, from 0.6% to 31.6% in the general population.^13^ Few studies report prevalence rates in the UK,^14,15^ but these studies included small samples and did not study a random sample of the population, possibly providing a biased view. Only one previous study has reported incidence rates in the UK,^16^ but this was carried out over 15 years ago. We are not aware of any literature reporting on the incidence of DD treatment per se. Reliable reports on prevalence and incidence of DD, and incidence of surgical intervention are relevant for the range of clinicians involved in management of DD, and for cost-effectiveness analyses of treatment modalities. They also provide vital information for commissioners planning the provision of services. In this cohort study, we used the UK Clinical Practice Research Datalink (CPRD) database combined with Hospital Episode Statistics (HES) Admitted Patient Care data to answer the following research questions: What are the prevalence and incidence of DD? What is the incidence of first surgical intervention for DD? What is the lifetime risk of surgical intervention after DD diagnosis?

## PATIENTS AND METHODS

In this population-based cohort analysis, we used CPRD data linked to HES data for evaluating prevalence and incidence of DD, incidence of first surgical intervention, and lifetime risk of first surgical intervention, using a multistate Markov model.

### Ethical Approval

This study was approved by the Independent Scientific Advisory Committee for Medicines and Healthcare Products Regulatory Agency Database Research (protocol 14_196R).

### Data Sources and Study Participants

We used data from the CPRD, a primary care database containing the anonymized health records of approximately 17.7% of the UK population,^17^ between January 1, 1995, and December 31, 2013, inclusive. We identified patients who had a diagnosis of DD recorded within this time, and who had linkage with HES. We used HES to derive information on surgical treatment. Because HES covers only England, only subjects from England were used in our analyses.

**Fig. 1.**
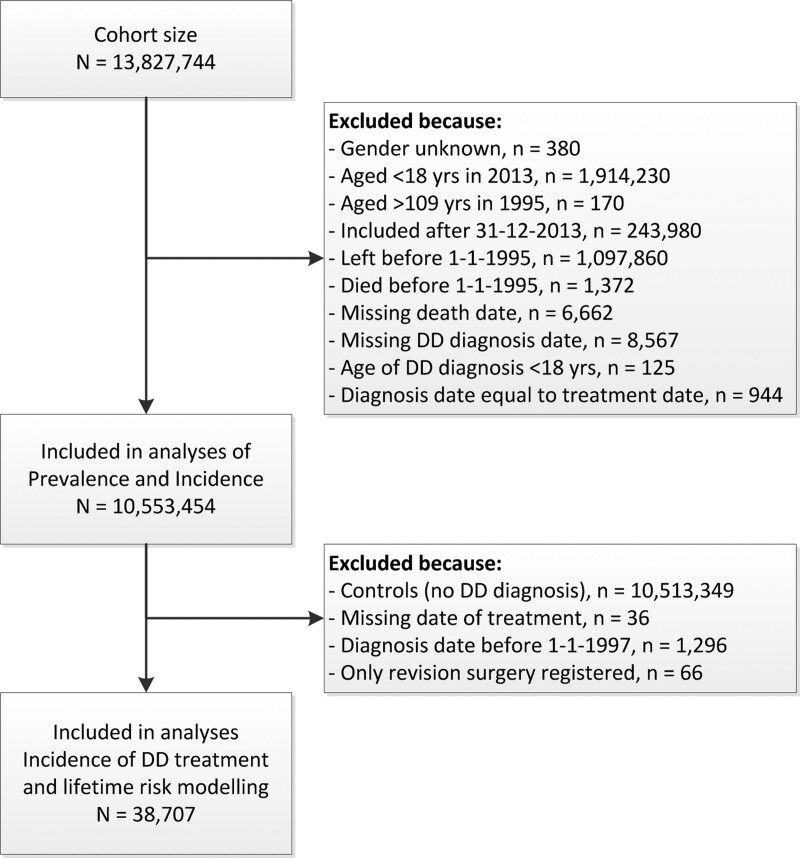
Flowchart of the selection process for obtaining the data sets used in the analyses.

We excluded subjects who did not have a recorded gender (Fig. 1). Subjects were removed from the study if they died or left their index CPRD-contributing research-quality general practitioner (GP) before 1995 for any reason. Any subject younger than 18 or older than 109 years was excluded, as were those with missing date of death. Those with a missing date of DD diagnosis or wrong diagnosis date (diagnosis date equal to treatment date) were excluded as well. Additional exclusion criteria were used for the analyses of lifetime risk of first surgical treatment, as subjects at risk are those diagnosed with DD. Therefore, subjects not diagnosed with DD were excluded, as were DD patients with a missing treatment date. For some of the treated DD patients, revision surgery was the only registered surgical intervention; these patients were excluded. Because the HES database started in 1997, those diagnosed before 1997 were also excluded from further analyses. We used a run-in period of 3 years; therefore, the results are presented starting from the year 2000.

### Outcomes

Patients with DD were identified according to a Read-code list defined independently by two clinically qualified researchers (R.Y.L.K. and D.F.), with consensus achieved by discussion with a third clinically qualified researcher (D.P.A.) if required. (**See Table, Supplemental Digital Content 1**, which shows the identification of DD cases and surgical interventions, http://links.lww.com/PRS/F639.)

In addition, we used linked HES data to identify patients who underwent first surgical treatment for DD within our study period. We classified procedures based on OPCS Classification of Interventions and Procedures version 4 codes (**see Table, Supplemental Digital Content 1**, http://links.lww.com/PRS/F639) as primary and secondary procedures for DD, where secondary procedures were revision operations performed after a primary procedure. Other parameters that we registered were year of birth, gender, GP region, diagnosis of diabetes (yes/no) and type, epilepsy (yes/no), and hypercholesterolemia (yes/no).

### Statistical Analyses

#### Prevalence and Incidence

Point prevalence of DD stratified on gender was calculated by creating a cohort of subjects included in CPRD at December 31, 2013. The number of DD cases was divided by the total size of the cohort at that time. Incidence of DD and incidence of first surgical intervention on a yearly basis was determined by creating cohorts of the population at risk according to year-specific exclusion criteria for each specific year. (**See Figure, Supplemental Digital Content 2**, which shows the selection process to obtain year-specific cohorts, http://links.lww.com/PRS/F640.) For each of the created cohorts, incidence was calculated by dividing the number of DD cases newly diagnosed or first surgically treated DD cases by the person-years at risk. Adjustment for differences in age distribution among the different cohorts was performed using direct standardization. As no change in gender distribution was observed across years, no standardization for gender was applied. The incidence of DD and the incidence of first surgical intervention were stratified on gender by different age categories by creating cohorts for each age category, and then determined as described above. As these analyses were split by age category, direct standardization was not applied.

#### Observed Risk of First Surgical Intervention for DD

Kaplan-Meier 1 − survival curves were plotted to evaluate observed risk of first surgical intervention for DD, with separate curves for gender, and age at diagnosis (categorized on quartiles).

#### Lifetime Risk of First Surgical Treatment for DD

For the estimation of lifetime risk of first surgical intervention after DD diagnosis, a multistate Markov model was used. (**See Figure, Supplemental Digital Content 3**, which shows statistical methods used for estimating lifetime risk of first surgical intervention for DD, http://links.lww.com/PRS/F641.) A hypothetical cohort of individuals entered the Markov model in the DD state. Every first surgical intervention in our hypothetical cohort is represented as a transition from the DD state to the surgical intervention state, and every death in our hypothetical cohort is represented as a transition from the DD state to the death state.

The transition probabilities of progressing from the DD state to the first surgical intervention state were estimated on the basis of a parametric survival model. The parametric survival model was estimated with time from diagnosis to first surgical intervention or censoring as the outcome variable. Alternative distributions for the parametric model were assessed by estimating it using the Weibull, exponential, log-linear, log-normal, and spline-based distributions, the latter with one, two, and three knots. The spline model with three knots was chosen based on the fit to the observed data and the plausibility of its extrapolation. (**See Figure, Supplemental Digital Content 4**, which shows the observed and estimated risk of first surgical intervention, http://links.lww.com/PRS/F642.) Age at diagnosis (continuous) and gender were included as covariates so that lifetime risk could be estimated for different values of diagnosis age and gender. We postulated that diagnosis year also might influence the chance of treatment; thus, we plotted the proportion of first surgical interventions in each year of diagnosis. No clear trend was visible, so year of diagnosis was not included in the model.

Transition probabilities of progressing from the DD state to the death state were derived from the UK life tables of the Office for National Statistics for the years 2013 to 2015.^18^ The model was run for different patient profiles to estimate the effect of specific patient characteristics. Uncertainty was added to the model by bootstrapping, deriving 99% confidence intervals from the results. Lifetime risk of first surgical intervention was calculated as the proportion of patients who progressed from the DD state to the first surgical intervention state, for each specific patient profile. An extensive description of the analyses to estimate lifetime risk is presented in **Figure, Supplemental Digital Content 3**, http://links.lww.com/PRS/F641.

All statistical analyses were done in RStudio version 1.1.383. Analyses were performed using packages epitools, mstate, mc2d, survival, survminer, flexsurv, bshazard, splines, and simPH. Packages ggplot2, ggalt, ggfortify, and gtools were used for data visualization.

## RESULTS

### Baseline Characteristics

Table 1 shows the demographics of our study population. Within our population of 10,553,454, we identified 38,707 cases of DD, of which 64.6% were men and 35.3% were women. Among those having the disease, male gender was more frequently observed in those with surgical intervention.

**Table 1.** Characteristics of the DD Cohort and Those Having First Surgical Treatment^a^

	DD Patients (%)	DD Patients Having First Surgical Treatment (%)
No.	38,707	4641
Gender		
Male	25,005 (64.6)	3606 (77.7)
Female	13,702 (35.4)	1035 (22.3)
Age at diagnosis, yr		
Median	63	64
IQR	55–71	56–70
Age at first treatment, yr		
Median	—	66
IQR	—	59–72
Region of GP		
North East	752 (1.9)	152 (3.3)
North West	4999 (12.9)	844 (18.2)
Yorkshire and the Humber	1689 (4.4)	291 (6.3)
East Midlands	1455 (3.8)	171 (3.7)
West Midlands	3512 (9.1)	627 (13.5)
East of England	3527 (9.1)	544 (11.7)
London	2766 (7.2)	346 (7.5)
South West	3458 (8.9)	651 (14.0)
South Central	4564 (11.8)	491 (10.6)
South East Coast	3781 (9.8)	524 (11.3)
Northern Ireland^b^	1107 (2.9)	—
Scotland^b^	3447 (8.9)	—
Wales^b^	3650 (9.4)	—
Diabetes	6557 (16.9)	692 (14.9)
Type 1	833 (12.7)	82 (11.9)
Type 2	5277 (80.5)	562 (81.2)
Missing	447 (6.8)	48 (6.9)
Epilepsy	751 (1.9)	102 (2.2)
Hypercholesterolemia	4326 (11.2)	485 (10.5)

IQR, interquartile range.

a There were no missing values in these variables, unless otherwise specified.

b Because information on surgical intervention was derived from HES, which covers only England, patients from Northern Ireland, Scotland, and Wales were excluded from the cohort that was used in the analysis of incidence of surgery, and lifetime risk of surgical intervention.

### Point Prevalence and Incidence of DD

**Fig. 2.**
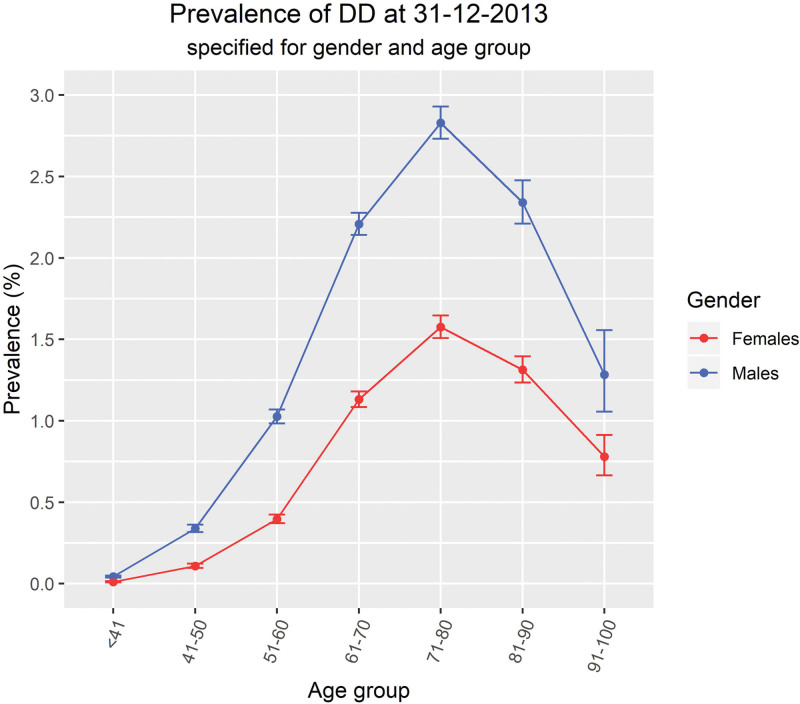
Point prevalence of DD in 2013 presented for gender with 99% confidence intervals.

The point prevalence of DD in 2013 was 0.672% (99% CI, 0.663 to 0.682). The prevalence was higher for men (0.884, 99% CI, 0.868 to 0.901) compared with women (0.469; 99% CI, 0.458 to 0.481). The prevalence of DD was highest among those aged between 75 and 85 years, both for men and for women (Fig. 2).

**Fig. 3.**
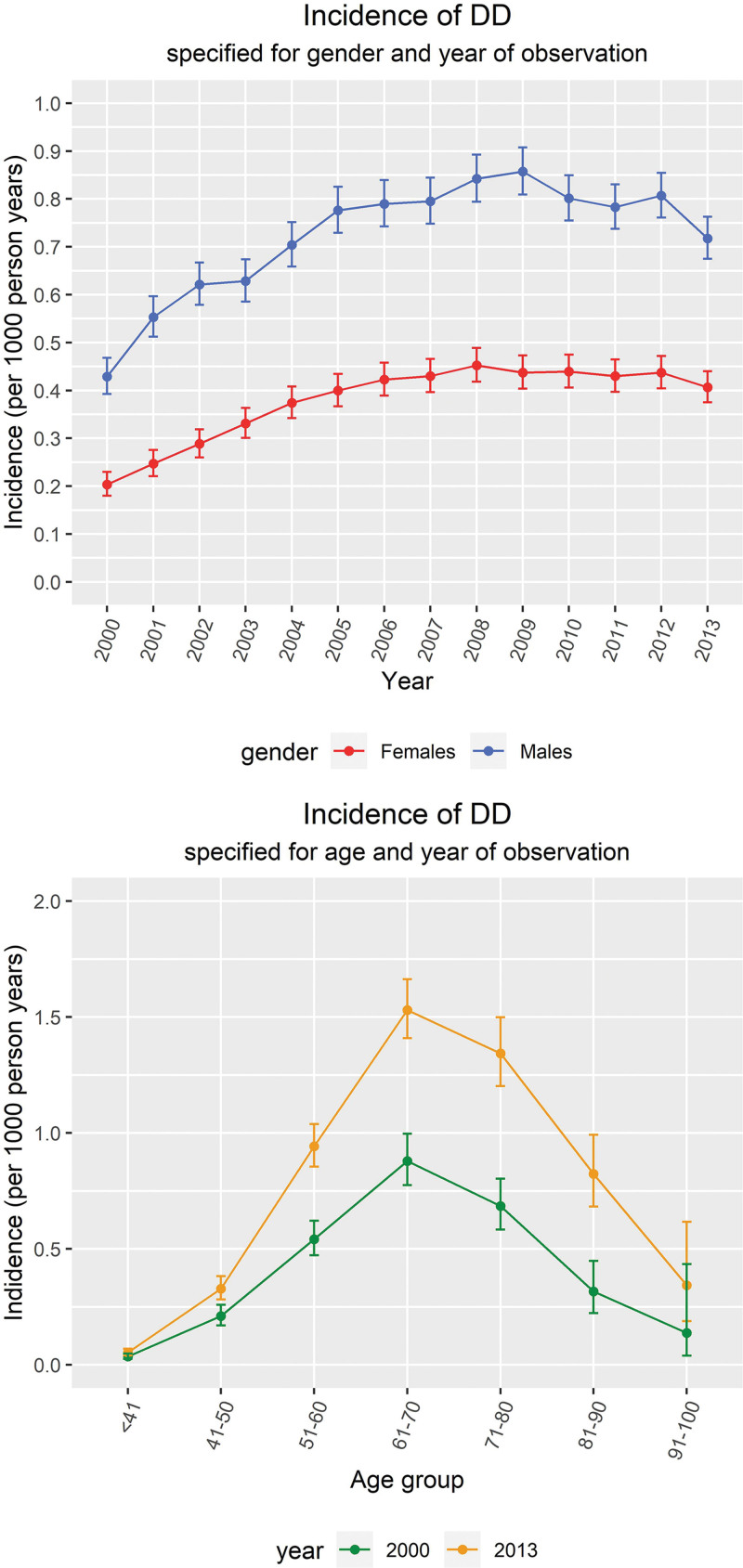
(*Above*) Incidence of DD specified for gender and year of observation with 99% confidence intervals. (*Below*) Incidence of DD specified for age and year of observation with 99% confidence intervals. Data before 2000 are omitted because of a run-in period of CPRD recorded diagnoses.

Incidence rates of DD increased during our study period, from 0.303 (99% CI, 0.282 to 0.325) per 1000 person-years in 2000 to 0.587 (99% CI, 0.559 to 0.616) per 1000 person-years in 2013, standardized for age. This increase was present in both men and women, although it was more pronounced in men (Fig. 3). Interestingly, the incidence of DD tends to stabilize around the year 2006, and even shows a decrease between 2006 and 2013 for men. Overall, the incidence in men was almost double the incidence in women. The highest incidence of DD is observed between 65 and 75 years of age.

### Incidence of First Surgical Intervention for DD

**Fig. 4.**
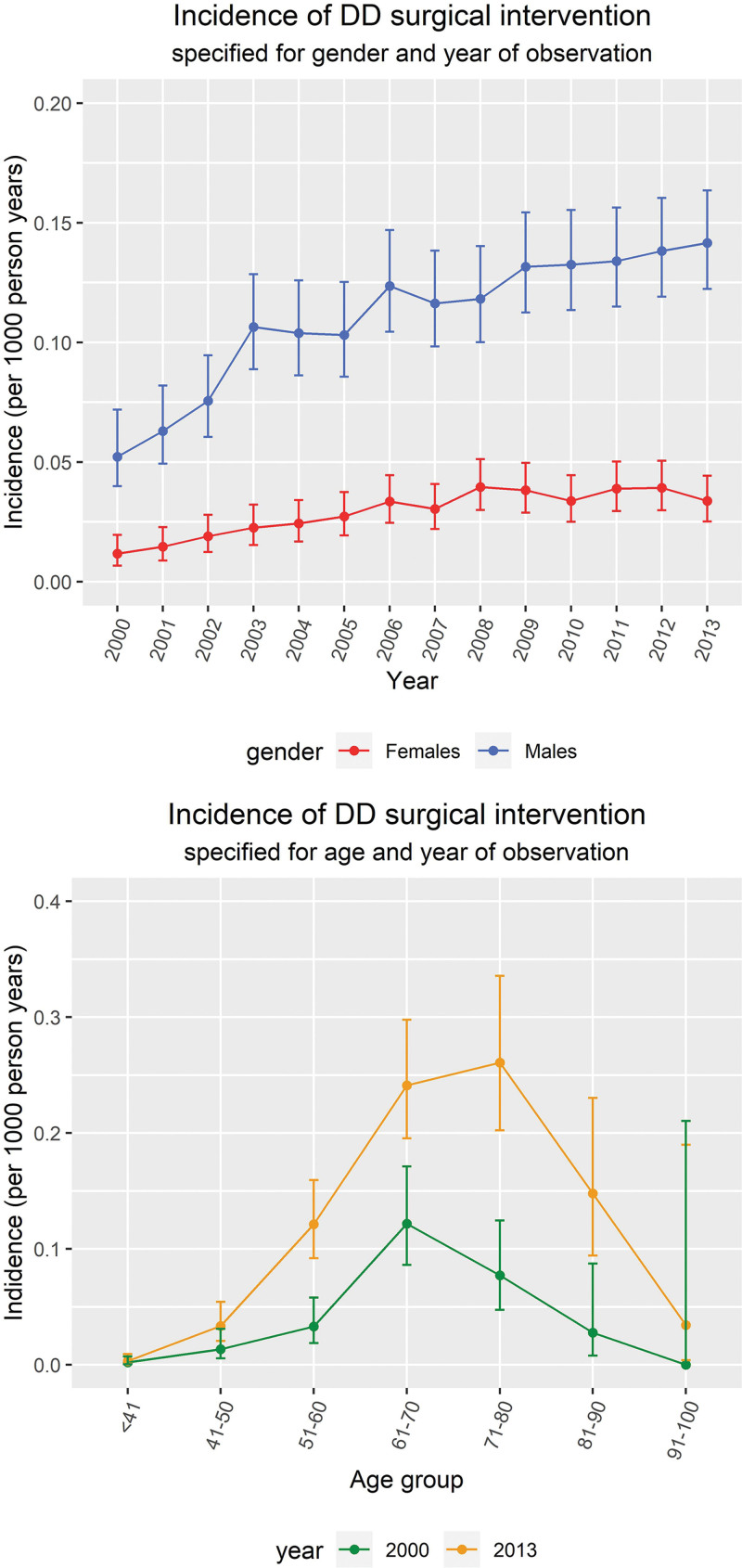
(*Above*) Incidence of first surgical intervention for DD specified for gender and year of observation with 99% confidence intervals. Data before 2000 are omitted because of a run-in period of HES. (*Below*) Incidence of first surgical intervention for DD specified for gender and age at diagnosis with 99% confidence intervals.

The incidence of first surgical intervention for DD mirrored the overall incidence of disease (Fig. 4), with an observed incidence of 0.294 per 1000 person-years (99% CI, 0.234 to 0.370) in 2000 and 0.878 per 1000 person-years (99% CI, 0.773 to 0.995) in 2013. The incidence for men was more than double the incidence for women, although for some years this difference was less pronounced. The incidence was highest among those diagnosed between 65 and 75 years of age, which was the same in 2013.

### Observed Risk of First Surgical Intervention for DD

We observed that men are more likely to undergo first surgical intervention than women. [**See Figure, Supplemental Digital Content 5**, which shows the observed risk of first surgical intervention (Kaplan-Meier 1 − survival curves), http://links.lww.com/PRS/F643.] The hazard ratio for men was 2.059 (95% CI, 1.920 to 2.208) compared with women. For the overall effect of age, a hazard ratio of 1.011 (95% CI, 1.009 to 1.014) was found, suggesting that the risk of treatment increases with increasing age of diagnosis. We indeed observed that those being diagnosed at age 70 or later have a high risk of being treated in the first year after diagnosis (**see Figure, Supplemental Digital Content 5**, http://links.lww.com/PRS/F643), higher than those diagnosed earlier in life. After this first year, the risk of surgical intervention decreases. This peak in risk of surgical intervention is less pronounced for those diagnosed earlier in life.

### Lifetime Risk of First Surgical Intervention for DD

**Fig. 5.**
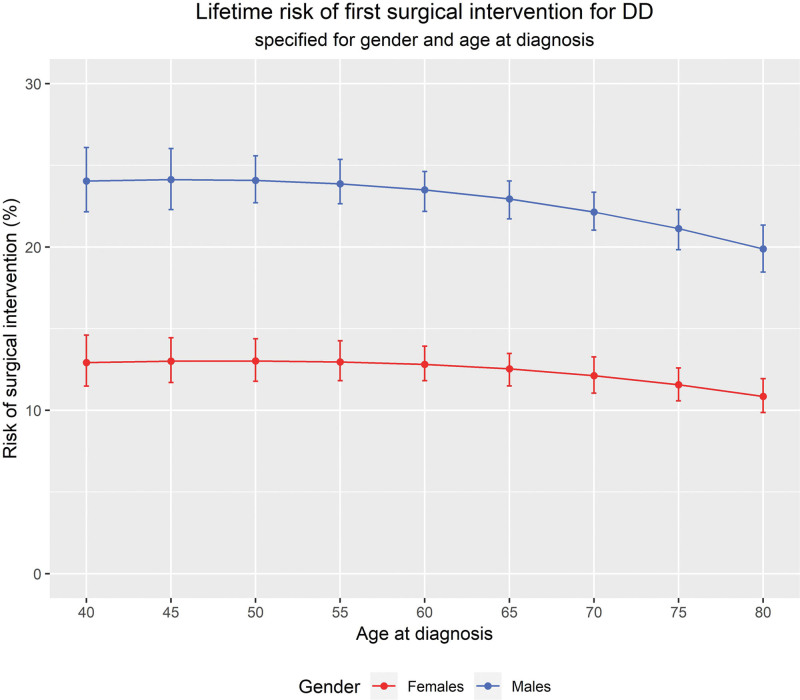
Lifetime risk of treatment for DD with 99% CIs, presented for men and women separately and by age of diagnosis.

Figure 5 shows the risk that patients presenting with DD to a GP will undergo surgical intervention over the course of their lifetime, specified for gender and age at diagnosis. Those diagnosed at an early age have the highest risk of getting treatment, although the risk of first surgical intervention is similar for diagnosis at ages 40 to 60. Men have almost double the risk of being treated compared with women. Overall, the risk of ever being treated is 23% (99% CI, 22 to 24%) in men and 13% (99% CI, 11 to 13%) in women diagnosed at 65 years of age, and stable across age groups.

## DISCUSSION

### Principal Findings

In this article, key concepts of the epidemiology of DD in the UK are presented. We report an overall prevalence of 0.7% and incidence of 0.59 per 1000 person-years in 2013. This means that in 2013, over 31,500 people in England were diagnosed with DD. We also observed that incidence of DD and incidence of first surgical intervention are increasing across years, indicating an increased burden to the health care system. Most importantly, we estimated that the lifetime risk that a male or female DD patient requires surgical intervention ever in his or her life is 23% and 13%, respectively, when diagnosed at age 65, showing only a very subtle decrease when diagnosed later in life.

### Findings in Relation to Other Studies

The prevalence found in our study is lower than the prevalence that some others report.^5,19,20^ This discrepancy is mainly caused by a difference in definition of diagnosis. In some prevalence studies, participants were prospectively examined for signs of DD, whereas in our study, only cases diagnosed by the GP were detected. Although this may have led to an underestimation of the true prevalence of DD in England, the prevalence rates reported in our study reflect the actual burden on the health care system.

The prevalence and incidence of DD that we report are slightly lower than the prevalence and incidence found in a database study conducted in Sweden,^21^ which might be explained by population differences, differences in the way the disease is registered, or by differences in definition of diagnosis. We observed that the incidence in men was almost twice that of women, which is consistent with other reports.^21^ The incidence peaks at 61 to 70 years of age, both in 2000 and 2013. This peak was also present in the Swedish population, although it occurred slightly later.^21^ In an earlier study of men from England and Wales, no peak was seen in incidence across age groups; incidence increased with age.^16^ Their study excluded data from women, explaining the higher reported incidence rates compared to our study. Moreover, we observed a large increase in incidence of DD diagnoses between 2000 and 2009. It is possible that this increase in incidence may not only represent an increase in the number of people affected, it may also reflect an increase in disease awareness.

We observed that the incidence of first surgical intervention for DD increased between 2000 and 2013. Once diagnosed, men were almost two times more likely to be treated than women. This is in line with previous findings, although in these studies a male-to-female ratio of 3:1 and 4:1 is reported.^11,22,23^ This predominance in men is in line with the clinical view of male gender being a risk factor for more aggressive disease.^24–26^ However, it may also reflect a gender-difference in referral strategy of GP, or a gender-difference in patient willingness to undergo surgical treatment. The incidence of first surgical intervention peaked at approximately 61 to 70 years of age in 2000, and a slight shift was observed with peak incidence between 71 and 80 years of age in 2013. The peak in incidence of first surgical intervention coincides with the observed peak in incidence of DD, which indicates that in patients who require surgery, treatment occurs shortly after diagnosis by the GP.

The lifetime risk of first surgical intervention is relatively stable for diagnosis ages 40 to 70. This may be explained by the fact that we included percutaneous needle fasciotomy as a surgical intervention, which is a minimally invasive treatment that is performed under local anaesthesia, and has no age-specific complication risks. It is likely that the distributions of different types of surgical interventions vary across age groups. The results of the observed risk of intervention seem contradictory to the estimated lifetime risk, as those diagnosed late in life had a higher risk of being treated. However, patients diagnosed later in life have less time to get treatment compared with patients diagnosed early in life, which explains why the lifetime risk is lower for those diagnosed later in life.

### Strengths and Limitations

Our study benefits from a large cohort size; over the period from January 1, 1995, to December 31, 2013, we have accumulated data of 10,553,454 patients with a median follow-up time of 9 years. To our knowledge, with inclusion of 38,707 DD patients, this is the largest cohort of DD patients described in the literature. The health care system in the UK covers all surgical procedures for DD, so no selection bias as a consequence of insurance status was expected. Our study has some limitations, as we have identified cases of DD using codes from GP databases. This means that lifetime risk of first surgical intervention is overestimated for the general population and underestimated for a clinical population. Furthermore, the use of GP diagnoses allows us to study the incidence and prevalence of diagnosed DD, but not the severity of disease. We were unable to study exact disease course in individual patients, although by using HES data we were able to use surgical intervention as a proxy for progression. We hypothesize that patients who have a diagnosis of DD in their GP history are more likely to have a severe form of DD; therefore, it is more likely that our incidence and prevalence rates underestimate true disease burden, particularly for subclinical cases. In addition, DD codes in the UK CPRD do not specify laterality of disease, so we were unable to determine whether repeated procedures for DD identified in HES were performed on the same side, or whether patients underwent intervention for both hands. Because of this limitation in coding, we only studied first-time surgical intervention for DD. Another limitation is that we did not have information on socioeconomic status, which might have influenced the estimated risk of surgical intervention. Furthermore, we did not include collagenase injections in our study, so the incidence of treatment and lifetime risk of treatment may be underestimated, although use of collagenase in the UK was low. As there are no UK-wide reports of clinical management of DD, it is difficult to specify the size of this underestimation. In our Markov model for estimating lifetime risk of treatment, we used ONS life tables to derive mortality risk. There are some indications that patients affected with DD have higher mortality risk,^27–29^ so our estimated lifetime risk of treatment may be overestimated.

### Meaning of Findings and Future Research

Our results show that DD is an important health condition in the older population, frequently requiring surgical intervention. Our results, combined with previous findings that DD often recurs after treatment,^12,30,31^ indicate that the burden of this disease is substantial. As there are no preventive treatments available at the moment, DD research should focus on the mechanism of disease to make a future treatment preventing progression available.

## ACKNOWLEDGMENT

This work was supported by the C.&W. de Boer foundation (to D.C.B.).

## Supplementary Material


