# Supplementary material for: Dupuytren Disease: Prevalence, Incidence, and Lifetime Risk of Surgical Intervention. A Population-Based Cohort Analysis
Source: Plast Reconstr Surg. 2022 Nov 22;151(3):581–91. doi: 10.1097/PRS.0000000000009919 (PMC9944385; doi:10.1097/PRS.0000000000009919)
Supplement: Supplementary file 1 [file prs-151-581-s001.pdf]

Identification of DD cases and surgical interventions

Diagnosis of DD		Surgical intervention for DD	
Read codes	***Read term***	***Read codes***	***Read term***
N236.00	Dupuytren's contracture	7H3A000	Dermofasciectomy
N236.11	Palmar fascia contracture	7H32800	Digital fasciectomy
N236700	"Dupuytren's dis, palm and finger(s), nodules, no contracture"	7H34011	Division of hand fascia
N236400	"Dupuytren's disease - finger(s), nodules with no contracture"	7H34000	Division of palmar fascia
N236300	Dupuytren's disease of finger(s)	7H32011	Dupuytren hand fasciectomy
N236500	"Dupuytren's disease of finger(s), with contracture"	7H34012	Dupuytren hand fasciotomy
N236000	Dupuytren's disease of palm	7H35700	Fasciotomy hand
N236600	Dupuytren's disease of palm and finger(s)	7H34z11	Fasciotomy NEC
N236800	"Dupuytren's disease of palm and finger(s), with contracture"	7H32012	Hand fasciectomy
N236100	"Dupuytren's disease of palm, nodules with no contracture"	7H32400	Limited palmar fasciectomy
N236200	"Dupuytren's disease of palm, with contracture"	7H32013	McIndoe radical palmar fasciectomy
		7H34300	Needle fasciotomy of hand
		7H32000	Palmar fasciectomy unspecified
		7H32700	Palmar fasciectomy using open palm technique
		7H32500	Radical palmar fasciectomy
		7H32900	Revision of digital fasciectomy
		7H32100	Revision of palmar fasciectomy
