# Supplementary material for: Dupuytren Disease: Prevalence, Incidence, and Lifetime Risk of Surgical Intervention. A Population-Based Cohort Analysis
Source: Plast Reconstr Surg. 2022 Nov 22;151(3):581–91. doi: 10.1097/PRS.0000000000009919 (PMC9944385; doi:10.1097/PRS.0000000000009919)
Supplement: Supplementary file 2 [file prs-151-581-s002.pdf]

Selection process to obtain year-specific cohorts.

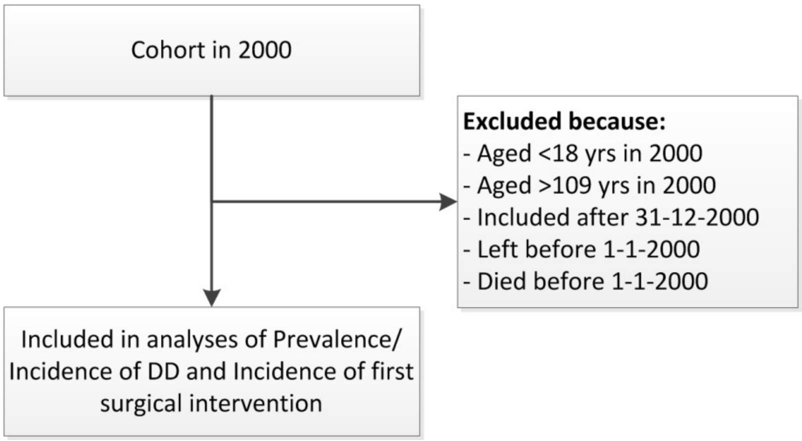

Example of the exclusion criteria used to obtain the dataset of the year 2000. For all other years, a similar selection process was used to obtain the datasets for analyses. DD: Dupuytren's disease.
