# Supplementary material for: Dupuytren Disease: Prevalence, Incidence, and Lifetime Risk of Surgical Intervention. A Population-Based Cohort Analysis
Source: Plast Reconstr Surg. 2022 Nov 22;151(3):581–91. doi: 10.1097/PRS.0000000000009919 (PMC9944385; doi:10.1097/PRS.0000000000009919)
Supplement: Supplementary file 3 [file prs-151-581-s003.pdf]

3. Statistical methods used for estimating Lifetime risk of first surgical intervention for DD

For the estimation of lifetime risk of first surgical intervention after DD diagnosis, a multistate **Markov model** was used (Figure I). This is a model characterised by multiple mutually exclusive states where the patients can be in (e.g. DD, surgery, death). These models are often used in decision analytics for estimating outcomes that cannot be (easily) observed in a study setting, for instance due to restrictions of time and resources, which would be the case when you want to study lifetime risk of a certain outcome.

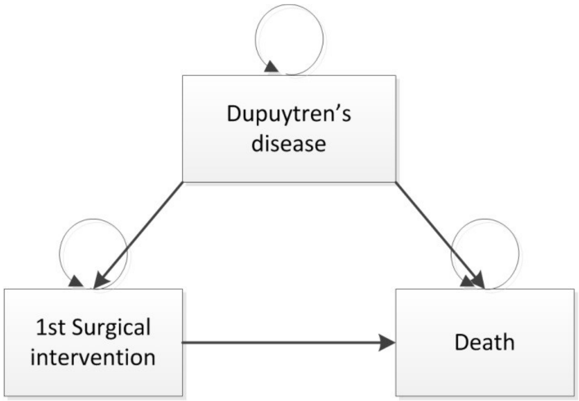

Figure I: Multistate Markov model, in which the straight arrows indicate the possible transitions, and the curved arrows indicate the possibility of staying in the same state.

Participants can move from one state to another in a certain amount of time, with a certain probability (**transition probability**). The transition probability is based on existing data, such as previous studies or known statistics. With this information, simulations can be done using the Markov model. In a simulation, a cohort of fake patients is created, that move through the states of the Markov model in a specific period of time called the **cycle**, for instance one year. In our case, a certain part of the fake DD patients got surgical treatment and a certain part died during the cycle. In each cycle, the number of patients in the DD state decreases, while the number of patients in the Surgery or Death state increases. This is in line with real life: after each year that passes, more and more DD patients will get surgery or die simply because of time passing by. Since the median age at diagnosis was 63 years and we were interested in lifetime risk, we decided to let the Markov model

run for 35 cycles, so that our fake cohort reached a median age of 98. After these 35 cycles, the number of fake patients being in the Surgery group, indicates the lifetime risk.

The model was run for different patient profiles to estimate the effect of specific patient characteristics. Lifetime risk of first surgical intervention after DD diagnosis was estimated for males and females, diagnosed at age 40, 45, 50, 55, 60, 65, 70, 75, and 80 years. Uncertainty was added to the model by **bootstrapping**, which is a method to estimate confidence intervals. Random samples from the data itself are drawn with replacement, on which the analyses are run. This is repeated a large number of times (in our case 1000 times), providing slightly different results. The spread in these results can be used for estimating confidence intervals. Lifetime risk of first surgical intervention was calculated as the proportion of patients who progressed from the DD state to the first surgical intervention state, for each specific patient profile.

All statistical analyses were done in RStudio version 1.1.383. Analyses were done using packages epitools, mstate, mc2d, survival, survminer, flexsurv, bshazard, splines, and simPH. Packages ggplot2, ggalt, ggfortify and gtools were used for data visualisation.
