# Supplementary material for: Dupuytren Disease: Prevalence, Incidence, and Lifetime Risk of Surgical Intervention. A Population-Based Cohort Analysis
Source: Plast Reconstr Surg. 2022 Nov 22;151(3):581–91. doi: 10.1097/PRS.0000000000009919 (PMC9944385; doi:10.1097/PRS.0000000000009919)
Supplement: Supplementary file 4 [file prs-151-581-s004.pdf]

Observed and estimated risk of first surgical intervention

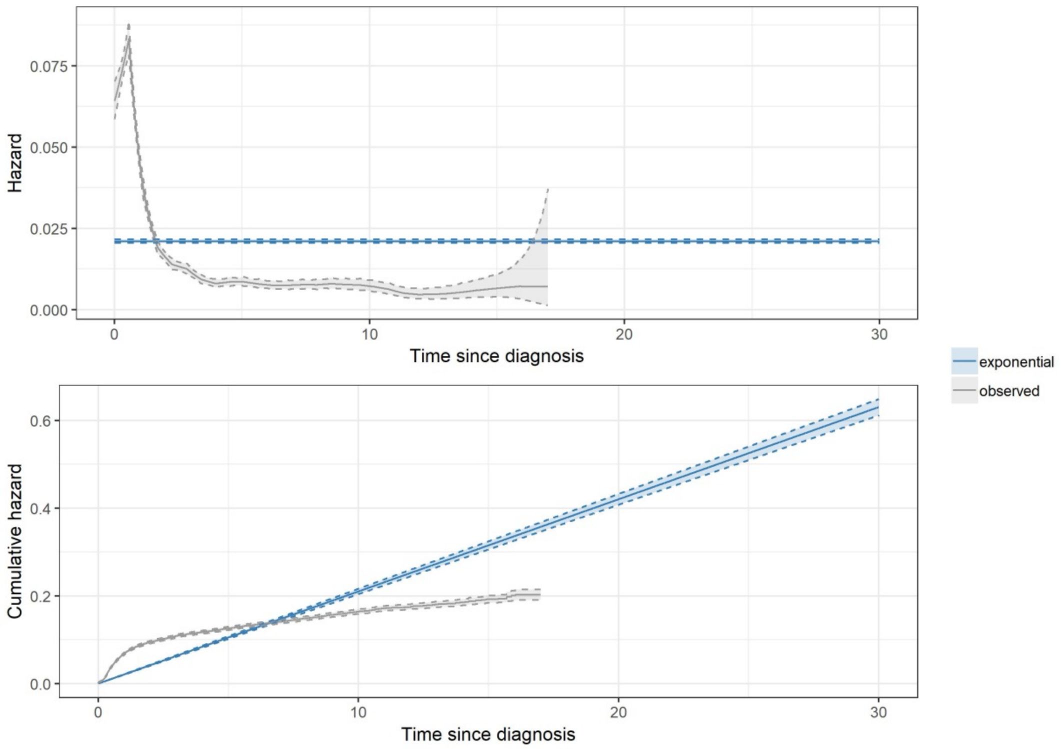

Figure I: Fit of model estimated using the exponential distribution, compared with observed risk.

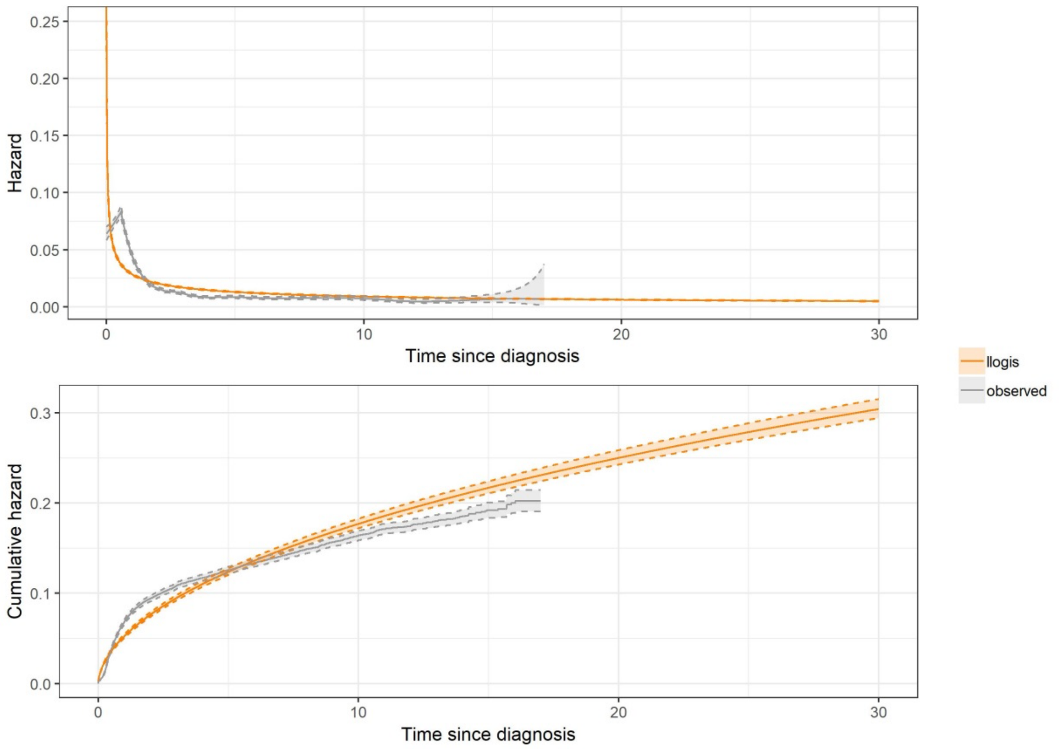

Figure II: Fit of model estimated using the log-logistic distribution, compared with observed risk.

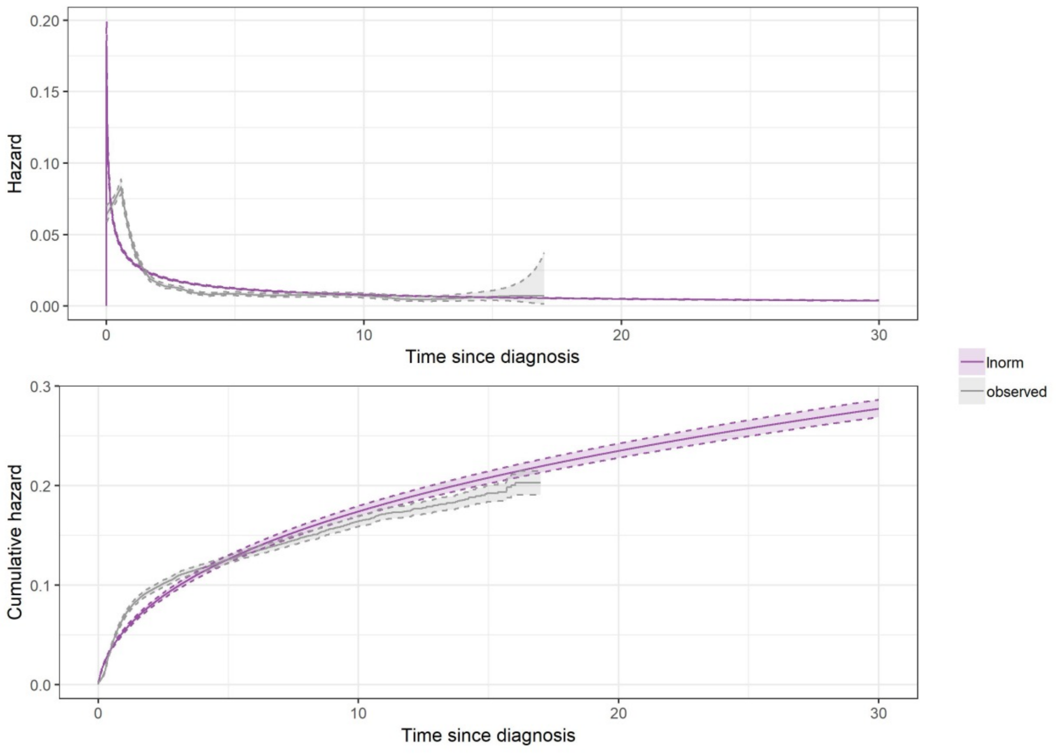

Figure III: Fit of model estimated using the log-normal distribution, compared with observed risk.

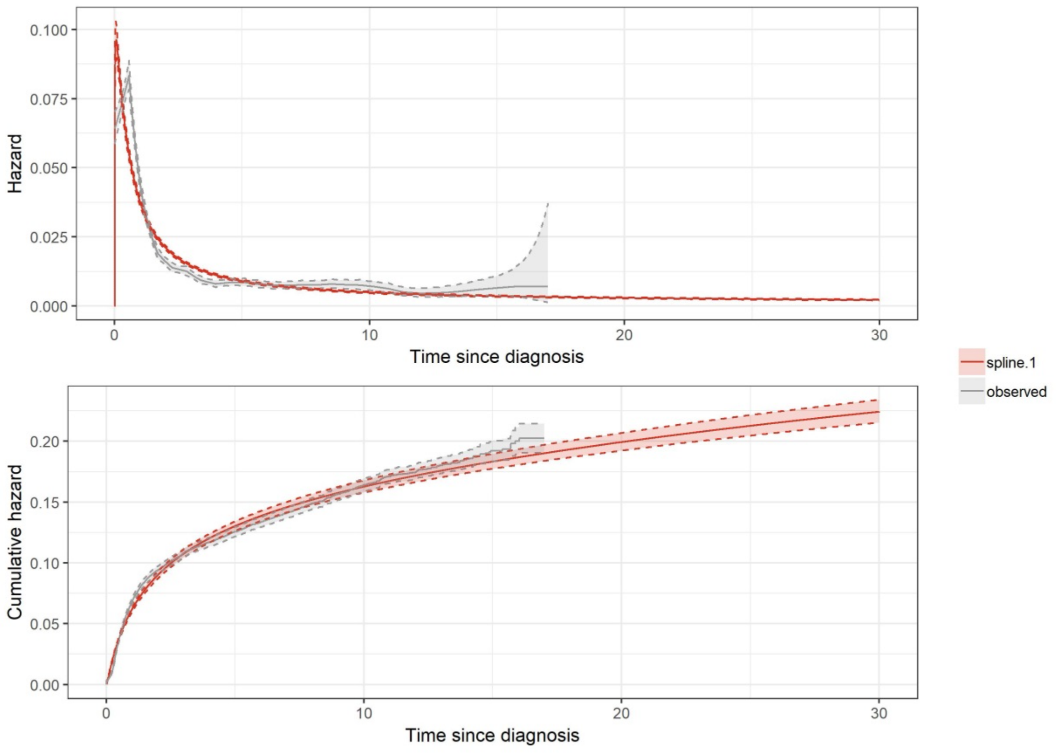

Figure IV: Fit of model estimated using the spline distribution with 1 knot, compared with observed risk.

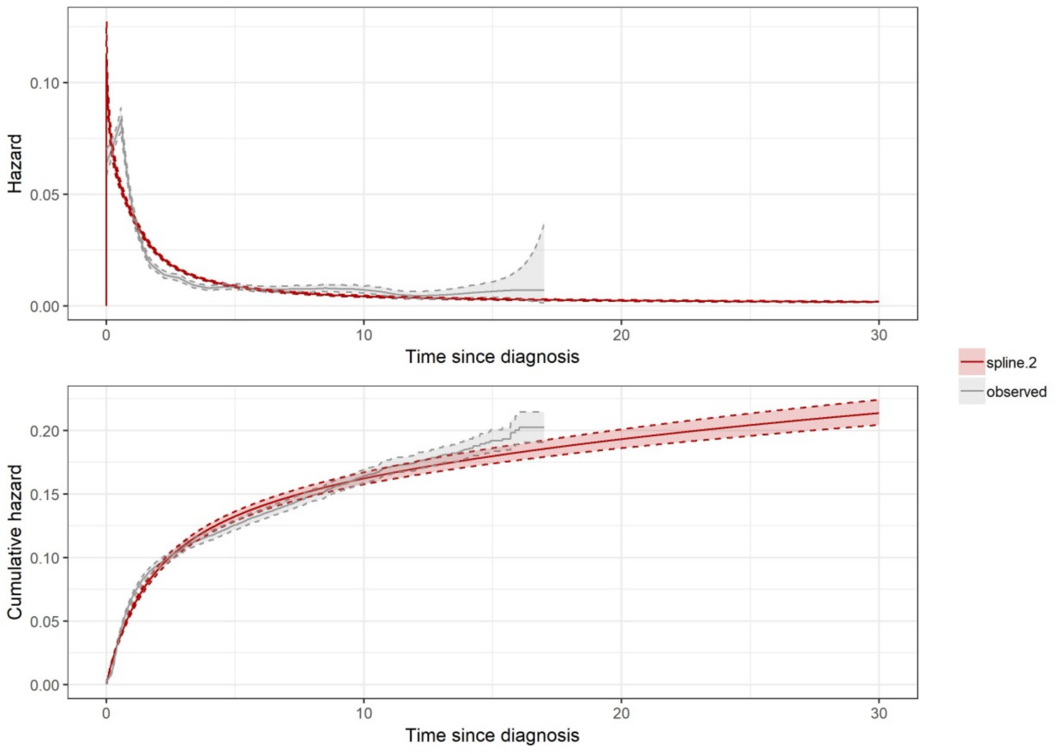

Figure V: Fit of model estimated using the spline distribution with 2 knots, compared with observed risk.

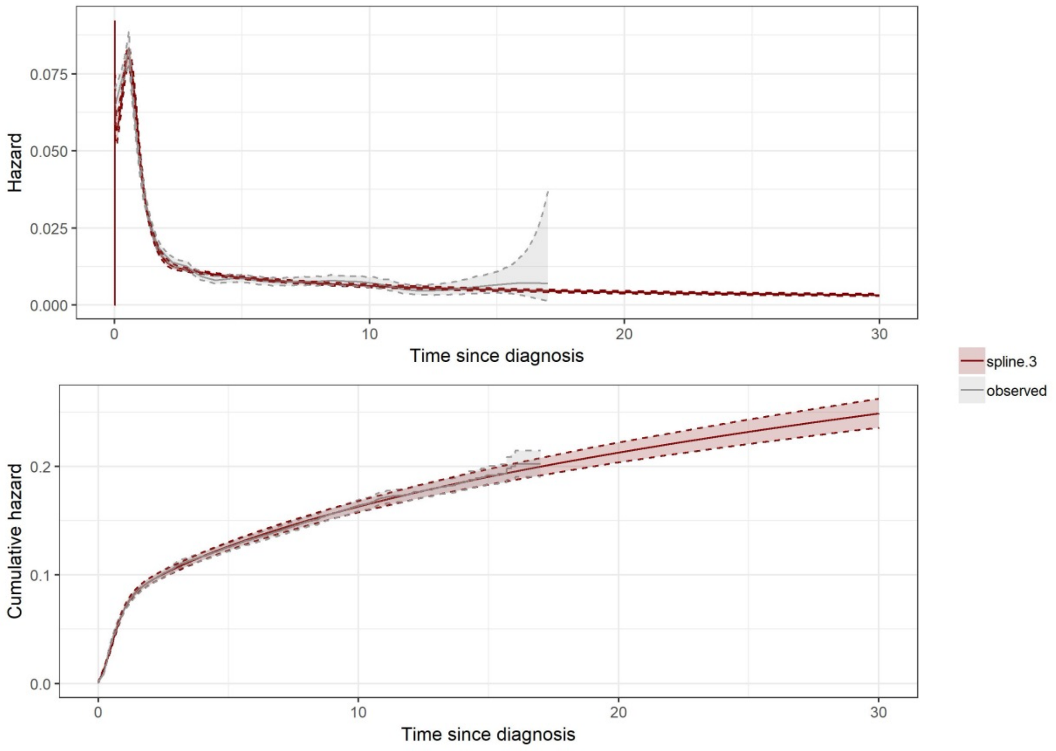

Figure VI: Fit of model estimated using the spline distribution with 3 knots, compared with observed risk.
