# Supplementary material for: Dupuytren Disease: Prevalence, Incidence, and Lifetime Risk of Surgical Intervention. A Population-Based Cohort Analysis
Source: Plast Reconstr Surg. 2022 Nov 22;151(3):581–91. doi: 10.1097/PRS.0000000000009919 (PMC9944385; doi:10.1097/PRS.0000000000009919)
Supplement: Supplementary file 5 [file prs-151-581-s005.pdf]

Observed risk of first surgical intervention – Kaplan-Meier 1-survival curves

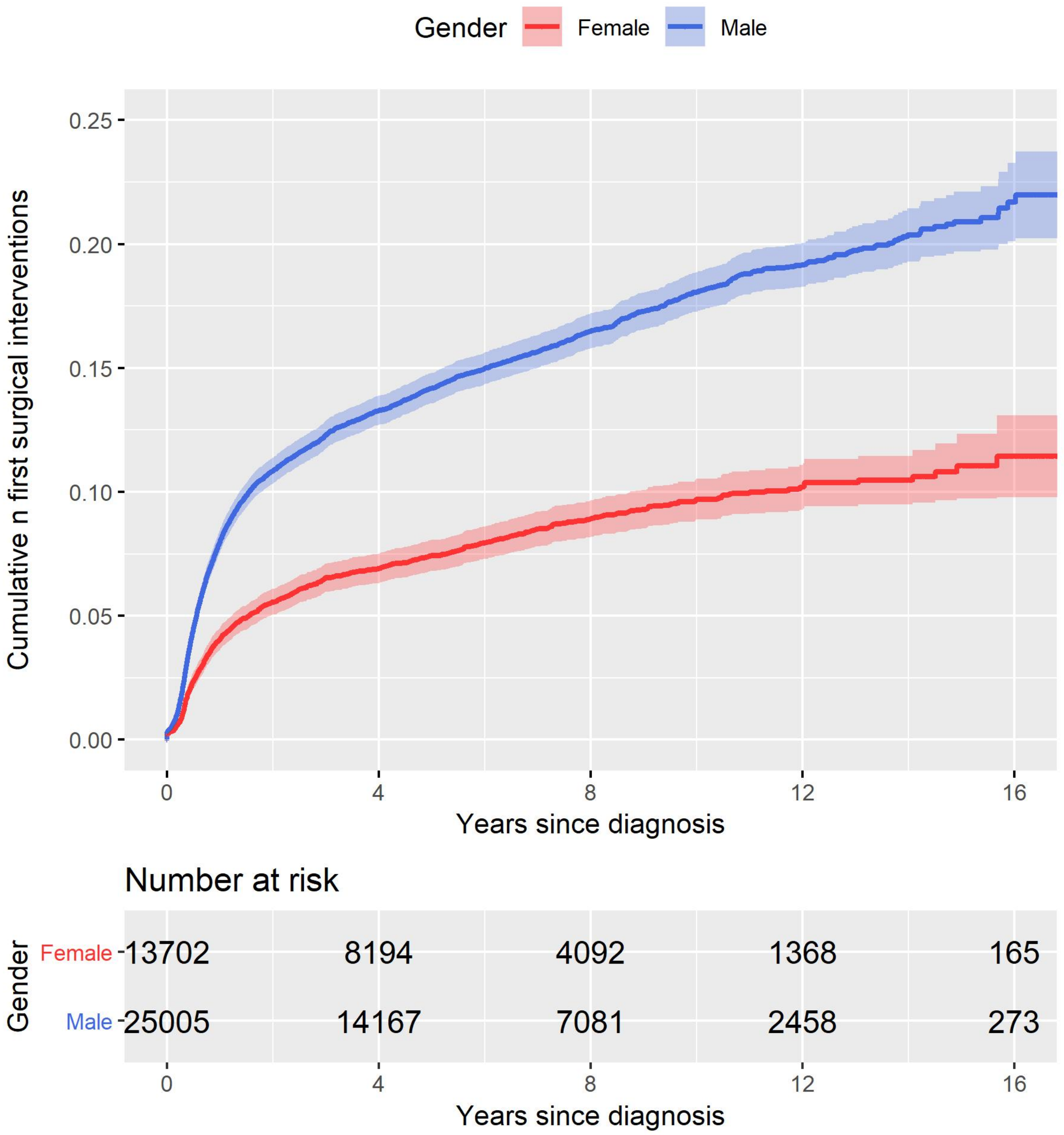

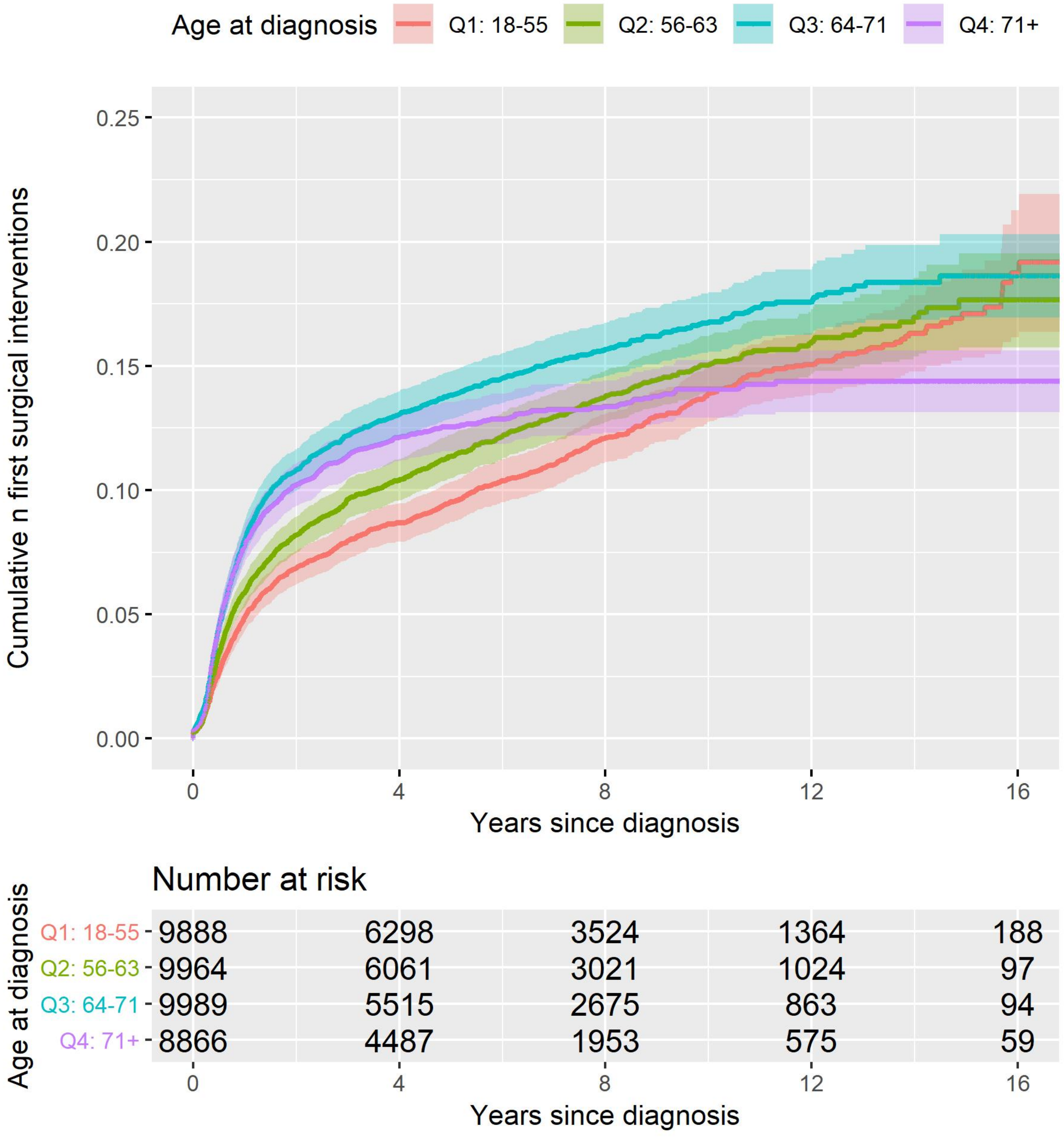
Age at diagnosis
Q1: 18-55
Q2: 56-63
Q3: 64-71
Q4: 71+
Cumulative n first surgical interventions
0.25
0.20
0.15
0.10
0.05
0.00
0
4
8
12
16
Years since diagnosis
Number at risk
Age at diagnosis
Q1: 18-55 9888 6298 3524 1364 188
Q2: 56-63 9964 6061 3021 1024 97
Q3: 64-71 9989 5515 2675 863 94
Q4: 71+ 8866 4487 1953 575 59
0
4
8
12
16
Years since diagnosis
